# The role of fingers in number processing in young children

**DOI:** 10.3389/fpsyg.2013.00488

**Published:** 2013-07-30

**Authors:** Anne Lafay, Catherine Thevenot, Caroline Castel, Michel Fayol

**Affiliations:** ^1^Centre de Recherche de l'Institut Universitaire en Santé Mentale de Québec, Université LavalQuébec, QC, Canada; ^2^Faculté de Psychologie et des Sciences de l'éducation, Department of Psychology, Université de GenèveGenève, Suisse; ^3^Centre National de la Recherche Scientifique, LAPSCO, Université Blaise PascalClermont-Ferrand, France

**Keywords:** numerical cognition, numerosity, preschoolers, non-symbolic representations, kindergartens

## Abstract

The aim of the present study was to investigate the relationship between finger counting and numerical processing in 4–7-year-old children. Children were assessed on a variety of numerical tasks and we examined the correlations between their rates of success and their frequency of finger use in a counting task. We showed that children's performance on finger pattern comparison and identification tasks did not correlate with the frequency of finger use. However, this last variable correlated with the percentages of correct responses in an enumeration task (i.e., Give-*N* task), even when the age of children was entered as a covariate in the analysis. Despite this correlation, we showed that some children who never used their fingers in the counting task were able to perform optimally in the enumeration task. Overall, our results support the conclusion that finger counting is useful but not necessary to develop accurate symbolic numerical skills. Moreover, our results suggest that the use of fingers in a counting task is related to the ability of children in a dynamic enumeration task but not to static tasks involving recognition or comparison of finger patterns. Therefore, it could be that the link between fingers and numbers remain circumscribed to counting tasks and do not extent to static finger montring situations.

## Introduction

Numerical symbols appear in a large variety of contexts such as price tags, shopping bills, phone numbers, street addresses, or arithmetic and mathematical problems. It is therefore important for researchers and practitioners to understand how numerical capacities develop and potentially dysfunction in children. Then, early identification of numerical difficulties has become a challenging and promising domain of research in order to construct and apply appropriate reeducation programs.

An early numerical ability that has received increasing attention from researchers in recent years is finger counting. As reminded by Dantzig (1962), Butterworth (1999) noted that “Whenever a counting technique, worthy of the name, exists at all, finger-counting has been found either to precede it or accompany it.” Then, finger counting constitutes an external aid to represent numbers, helps keeping track of number words in counting and sustains the comprehension of the 10-base numerical system as well as the realization of basic arithmetic operations. Due to the amount of activities based on finger counting, it is logically considered to play an important role in numerical capacities.

The relationship between fingers and numerical representations has been established in several studies involving children with manual difficulties. Arp and Fagard (2001) showed that counting difficulties in children with cerebral palsy depend on the severity of their visual-manual coordination deficits. It has also been shown that children with dyspraxia present a delay in mathematical acquisition, which could be due to their difficulties in pointing at objects and would, in turn, prevent them from counting collection appropriately (Lecointre et al., 2005). If, in those studies, the difficulties encountered by children result from visuo-manual coordination impairments, more recent studies isolate the role of manual deficits by assessing numerical abilities in children without coordination problems. Indeed, Thevenot and Fluss (2012) showed that children with congenital hemiplegia, who present difficulties in using one of their hands, also exhibit difficulties in symbolic numerical tasks. The relationship between numbers and fingers has also been well documented outside the field of neuropsychology. Fayol et al. (1998) showed that finger recognition performance in 5–6-year-old children is a better predictor of arithmetical performance one year later than more classical tests of intelligence such as Goodenough's draw-a-man test. This held true even three years later (Marinthe et al., 2001). Moreover, Costa et al. (2011) showed that finger gnosia performance is lower in children with mathematical difficulties than in children without difficulties. These results are coherent with those of Noël (2005) who revealed a positive correlation between finger gnosia and numerical performance in children at the beginning of Grade 1 (i.e., 6–7-year-old children). Gracia-Bafalluy and Noël (2008) even suggested that training finger gnosia may generalize to untrained numerical performance (but see Fischer, 2010).

A simple explanation of the relationship between numbers and fingers is given by the proximity of the brain areas devoted to their mental processing. This proximity has been suspected for the first time following Gerstmann's description (1940) of a series of patients who presented strange concomitant symptoms of agraphia, spatiotemporal confusion, finger agnosia, and acalculia. This syndrome, now known as the Gerstmann syndrome, has also been identified in children (Kinsbourne et al., 1963). Later on, neuro-imaging studies confirmed Gerstmann's intuition showing that the parietal lobe and the left precentral gyrus are involved both in numerical processing and finger gnosia (Di Luca et al., 2006; Sandrini and Rusconi, 2009). Therefore, a lesion of the left parietal lobe can affect both representations of fingers and representations of numerosities. This was also nicely confirmed by a rTMS study showing that an angular gyrus stimulation generates disruptions in tasks involving finger and numerical representations (Rusconi et al., 2005).

Going further than a mere explanation of the relationship between numbers and fingers by the proximity of the brain areas devoted to these activities, a functionalist interpretation postulates that finger counting constitutes the basis for future numerical abilities (see Fayol and Seron, 2005 for a review). Within this interpretation, the link between finger gnosia and numerical abilities is well explained by Reeve and Humberstone (2011) who showed that finger gnosia abilities change in the early school years and that these changes are associated with the ability to use fingers to aid computation. Furthermore and in accordance with theories of embodied cognition (Barsalou, 1999), Domahs et al. (2008) observed children's errors in addition and subtraction problems and showed that split-five errors were over-represented and above chance level. The authors concluded that mental representations of numbers that inherit sub-base five properties are built up and internalized during childhood. Interestingly, Domahs et al. (2010) also showed the influence of the sub-base five on numerical representations in adults. More generally, within the “manumerical” hypothesis (Fischer and Brugger, 2011), finger-based representations of numbers are seen as the result of an integration of multi-modal input during early finger counting and finger calculation in childhood and the later offline simulation of the according motor programs (Moeller et al., 2012). Moeller et al. (2012) even suggest that finger-based representations of numbers are activated automatically whenever a number is encountered. Also in line with the functionalist interpretation, Andres et al. (2007) suggested that finger use in numerical tasks could constitute a transition between the non-symbolic and symbolic systems and would therefore determine later performance in representation and manipulation of numbers in a pure symbolic format. The authors confirmed their proposition by showing corticospinal excitability of the muscles of the hand in silent numerical tasks in adults. This was interpreted as evidence for childhood reminiscence of finger use for the representation of number words. As noted by Andres et al. (2008), fingers may be the “missing tool” between non-symbolic and symbolic numerosities involved in arithmetic.

Therefore, the fact that finger use in counting shapes numerical mental representations seems well supported in the literature. However, whether or not finger counting is a necessary tool for the development of these representations, and consequently numerical abilities, is still open for debate (Plaisier and Smeets, 2011). As noted recently by Crollen et al. (2011b), the functional hypothesis would lead to the prediction that, during the first developmental stages, children should be more accurate to represent numerosities with their fingers than with number words. As a matter of fact, no such data is available so far in the literature. On the contrary, Nicoladis et al. (2010) showed that 2–5-year-old children performed equally bad when presented either with hand shape or number words and asked to put the corresponding number of toys in a box. Moreover, 4 and 5-year old children perform actually better with words than hand shape. Another interesting result that questions the role of fingers in shaping numerical abilities has been recently reported by Crollen et al. (2011a) who demonstrated that blind children use finger-counting strategies less often than sighted children. Still, blind and sighted children achieve similar level of performance in enumeration tasks. Crollen et al. concluded that fingers are a useful rather than necessary tool for the development of counting abilities.

These series of results question the necessity of finger use in the development of numerical abilities and suggest that further investigation is needed in order to determine the precise role of fingers in number processing. This is the aim of the present study. If finger use is indeed a useful step that constitutes a transient developmental stage between non-symbolic and symbolic numerical abilities, children who use their fingers less frequently should be those children with the poorer numerical performance. However and furthermore, if finger use is a necessary step in the numerical developmental course, children who do not use their fingers should not be able to succeed in symbolic numerical tasks. In order to verify this assumption, we assessed 4–7-year-old-children's performance on their spontaneous use of fingers in a counting task, on finger numerical pattern recognition and on an enumeration task. Seldom reliance on fingers for the counting task should be associated with poorer performance in finger pattern comparison and identification, and, in turn poorer performance in a “Give-me N” task (i.e., enumeration task).

The task that we developed in order to determine whether children use their fingers in a counting task is original and allows us to determine precisely whether finger use in numerical tasks is a strategy that belongs to children's repertoire. Children had to determine the total number of pictures in a collection presented in front of them on a table. They were asked to name the picture one by one, and, just after, they had to give the cardinal of the collection. Because, in this task, the phonological loop is blocked by picture naming (Baddeley, 1986), the best strategy in absence of other external aids is to keep track of the number of pictures on fingers. Of course, it was never mentioned to children that they had to (or even could) use their fingers to perform the task. Therefore, children who implemented the finger strategy did it spontaneously, without any constraint or insight from the experimenter. We think that this task is a better way to assess finger use in numerical activities than a mere observation of children's behavior during calculations. Indeed, when children do not use their fingers to solve arithmetic problems, it is impossible to determine whether they do not need them any longer or whether they never have resorted to them. On the contrary, in the picture counting task, fingers are still required to succeed in the task. Then, children who do not use them are necessarily children for whom this strategy is not available.

## Methods

### Participants

Sixty normally developing children took part in this experiment. Twenty of them were preschoolers aged between 4 and 5 years (*M* = 4.7, *SD* = 0.31; 9 females; 18 right-handed). Twenty of them were kindergarten children aged between 5 and 6 years (*M* = 5.6, *SD* = 0.29; 9 females; 17 right-handed). The 20 remaining children were in Grade 1 and were aged between 6 and 7 years (*M* = 6.7, *SD* = 0.29; 11 females; 19 right-handed). Children did not present any developmental disorders or disabilities.

### Materials and procedure

#### Spontaneous use of fingers in counting

As already mentioned above, children had to determine the total number of pictures in a collection presented in front of them on a table. They were asked to name the picture one by one, and, just after, they had to give the cardinal of the collection. We selected twenty pictures that lead to 100% of correct recognition in 4-year-old children (BD2I, Cannard et al., 2006). Children were presented with three small collections from 1 to 5 pictures, three medium collections from 6 to 10 pictures and three large collections from 11 to 15 pictures. For each child, the specific numerosities that were selected within each of the collection size were kept for the Give-*N* task (e.g., 2, 3, 5 for small; 6, 7, 10 for medium and 11, 14, 15 for large collections). Whether or not children used their fingers during the task, and whether or not they succeeded in giving the correct number of pictures presented constituted our two dependent variables of interest. The use of fingers was coded by the experimenter instantly during testing.

#### Finger numerical pattern recognition

Finger numerical pattern recognition was assessed with a comparison and an identification task. The materials we used for both tasks was adapted from Noël (2005; see also Gracia-Bafalluy and Noël, 2008).

***Comparison of finger numerical pattern.*** Sixteen different pictures representing one hand with one to four raised fingers were used in this task. Half of them represented right hands, while the other half represented left hands. More importantly, half of the pictures corresponded to canonical numerical finger patterns (e.g., culturally, raising the thumb, the index, and the middle finger represents 3 in France) and the other half to non-canonical patterns.

Twenty-four trials were constructed using those pictures. A trial corresponded to two pictures presented on screen at the same time. A third of the trials were composed of two pictures representing canonical patterns, another third of two pictures representing non-canonical patterns and a last third mixing canonical and non-canonical patterns (see Table A1). By pressing a key, children had to decide as quickly as possible if the two pictures showed the same number of raised fingers. Half of the trials required a “Yes” response and the other half a “No” response. Each trial was preceded by a fixation cross presented for 500 ms and the picture was displayed on screen until the answer was given. Four warm-up trials were presented before the experimental phase. Accuracy and reaction times were recorded by the computer.

***Identification of finger numerical pattern.*** In addition to the 16 pictures used in the previous task, 12 pictures representing two hands with six to nine raised fingers were added to the materials. The four canonical patterns corresponding to 6, 7, 8, and 9 were presented and, in order to increase the number of trials, two different non-canonical patterns were constructed for each numerosity (see Table A2). This resulted in twenty-eight experimental trials (i.e., 16 pictures representing one hand and 12 pictures representing two hands), which were preceded by three warm-up trials.

Children were asked to determine as quickly as possible the number of raised fingers on the pictures and the experimenter pressed a key as soon as participants uttered their answer. Reactions times were recorded by the computer, whereas errors were written down by the experimenter.

#### Enumeration task (give-N task)

Children had to give 1–15 tokens to the experimenter. The exact same numerosities than in the “Spontaneous use of fingers in counting” task were used here. As already explained, there were three trials per numerosity size (i.e., small numerosity: 1–5 tokens; medium numerosity: 6–10 tokens and large numerosity: 11–15 tokens). Children succeeded the task when they precisely took the number of tokens asked by the experimenter from a stack of tokens displayed in front of them.

## Results

In order to have a clear picture of children's behavior in the different tasks, an ANOVA will be performed for each of them. Moreover and in order to test our predictions, a correlation analysis between the frequency of spontaneous use of fingers and the other tasks will be reported for each of the tasks. Finally, in order to verify whether numerical abilities are really related to the use of fingers rather than to natural development, additional analyses will be conducted with the age of children as a covariate for each of the significant correlations.

### Spontaneous use of fingers in counting

#### Percentages of spontaneous finger use

Overall, 22 children out of 60 (36%) used their fingers at least once in the picture counting task. The number of children using their fingers increased as a function of age with only 1 preschooler (5%), 3 kindergartens (15%), and 18 children out of 20 in Grade 1 (90%) using them. For the sake of precision, the following ANOVA was carried out on a trial by trial basis and not on the rough percentages of children who used their fingers.

Because our dependent variable was binary, we applied an arcsin transformation to our data before carrying out the 3 (School level: Preschoolers, Kindergartens, and Grade 1) × 3 (Numerosities: Small, Medium, and Large) ANOVA on the transformed data (Table 1). Fingers were used in 29% of the trials and, in accordance with the previous results, the percentage of trials wherein fingers were used varied as a function of school level, *F*_(2, 57)_ = 30.21, η^2^_*p*_ = 0.51, *p* < 0.001. Children in Grade 1 used their fingers in 70% of the trials, whereas kindergartens and preschoolers used their fingers in only 12 and 5% of the trials, respectively. The percentages of trials wherein fingers were used also increased as a function of numerosities, *F*_(2, 114)_ = 18.02, η^2^_*p*_ = 0.24, *p* < 0.001 (17, 34, and 35% for small, medium, and large numerosities, respectively). Moreover, there was an interaction between the two factors, *F*_(4, 114)_ = 10.11, η^2^_*p*_ = 0.26, *p* < 0.001, showing that the effect of school level increased with numerosities.

**Table 1:** **Percentages of spontaneous finger use in the counting task, as a function of school level and numerosities**.

	**Small**	**Medium**	**Large**
First graders	41.67	83.33	85.00
Kindergartens	5.00	15.00	15.00
Preschoolers	5.00	5.00	5.00

#### Percentages of correct responses in the counting task

As in the previous analysis, an arcsin transformation was applied to the percentages of correct responses in the counting task and an ANOVA with the same design as before was carried out on the transformed data (Table 2). The percentages of correct responses increased as a function of school level, *F*_(2, 57)_ = 29.81, η^2^_*p*_ = 0.51, *p* < 0.001. First graders were more successful (78%) than kindergartens (43%), and preschoolers (32%). Furthermore, the percentages of correct responses decreased as a function of numerosities, *F*_(2, 114)_ = 98.49, η^2^_*p*_ = 0.63, *p* < 0.001 (with 87, 38, and 28% for small, medium, and large numerosities, respectively). Moreover, there was an interaction between the two factors, [*F*_(4, 114)_ = 7.89, η^2^_*p*_ = 0.22, *p* < 0.001] showing that the effect of school level increased with numerosities.

**Table 2:** **Percentages of correct responses in the counting task, as a function of school level and numerosities**.

	**Small**	**Medium**	**Large**
First graders	95.00	68.33	70.00
Kindergartens	90.00	28.33	10.00
Preschoolers	75.00	18.33	3.33

Finally, a correlational analysis between the transformed percentages of spontaneous use of fingers in the counting task and the transformed percentages of correct responses in this task revealed that these two variables were positively related (*r* = 0.74, *p* < 0.001). This held true when the age of children was entered as a covariate in the analysis (*r* = 0.51, *p* < 0.001). These results suggest that using fingers in the picture counting task is a good strategy that helps children keeping track of the number of pictures named. This is largely confirmed by a more descriptive observation showing that children who used their fingers in the task succeeded in 73% of the trials that were constructed with large numerosities whereas children who did not use their fingers succeeded in only 2% of the trials.

### Finger numerical pattern recognition

#### Comparison of finger numerical pattern

In order to draw our conclusions from a reliable measure and to eliminate any speed/accuracy trade-off effects, composite *Z* scores between accuracy and reaction times were calculated for each participant (see Table 3 for mean accuracy and RTs). Then, a 3 (School level: Preschoolers, Kindergartens and First graders) × 3 (Configuration: Canonical vs. Non-canonical vs. Mixed) ANOVA with the first factor as a between measure and the second factor as a repeated measure was performed on composite scores in the comparison of finger numerical pattern task.

**Table 3:** **Percentages of correct responses and reactions times (in ms) in the comparison of finger numerical pattern task as a function of school levels and finger configurations**.

	**Non-canonical**		**Mixed**		**Canonical**	
	**Percentages**	**RTs**	**Percentages**	**RTs**	**Percentages**	**RTs**
First Graders	91.25	2719	88.13	2574	92.50	2289
Kindergartens	85.00	2626	81.88	2802	88.13	2000
Preschoolers	83.75	3088	72.50	3274	80.63	2837

First graders and kindergartens performed better than preschoolers [*F*_(2, 57)_ = 6.51, η^2^_*p*_ = 0.19, *p* = 0.003]. Moreover, children were more successful when comparing canonical than non-canonical configurations [*F*_(1, 57)_ = 17.97, η^2^_*p*_ = 0.24, *p* < 0.001] or mixed configurations [*F*_(1, 57)_ = 8.84, η^2^_*p*_ = 0.13, *p* = 0.004]. There was no difference between these last two conditions [*F*_(1, 57)_ = 2.69, *p* = 0.11]. Moreover, there was no interaction between these two factors (*F* < 1). Finally, there was no correlation between the transformed percentages of spontaneous use of fingers in the counting task and the performance in the comparison of finger numerical patterns (*r* = −0.18, *p* > 0.05).

#### Identification of finger numerical pattern

As for the previous analysis, composite *Z* scores between accuracy and reaction times were calculated for each participant (see Table 4 for mean accuracy and RTs). A 3 (School level: Preschoolers, Kindergartens, and First graders) × 2 (Configuration: Canonical vs. Non-canonical) ANOVA with the first factor as a between measure and the last factor as a repeated measure was performed on the composite *Z* scores in the identification of finger numerical pattern task.

**Table 4:** **Percentages of correct responses and reaction times (in ms) in the identification of finger numerical pattern task as a function of school levels and finger configurations**.

	**Canonical**		**Non-canonical**	
	**Percentages**	**RTs**	**Percentages**	**RTs**
First graders	98.13	2407	90.63	3175
Kindergartens	87.81	3269	82.50	4047
Preschoolers	72.19	4423	68.44	5189

First graders performed better than kindergartens [*F*_(1, 57)_ = 11.56, η^2^_*p*_ = 0.17, *p* = 0.001], who, in turn, performed better than preschoolers [*F*_(1, 57)_ = 17.77, η^2^_*p*_ = 0.24, *p* < 0.001]. Moreover, canonical configurations led to better performance than non-canonical configurations, *F*_(1, 57)_ = 18.83, η^2^_*p*_ = 0.25, *p* < 0.001. However, planned comparisons showed that this was true only for first graders and kindergartens [*F*_(1, 57)_ = 12.17, η^2^_*p*_ = 0.18, *p* < 0.001 and *F*_(1, 57)_ = 9.06, η^2^_*p*_ = 0.14, *p* = 0.004, respectively] but not for preschoolers [*F*_(1, 57)_ = 1.04, *p* = 0.31].

Finally, a correlational analysis between the transformed percentages of spontaneous use of fingers in the counting task and performance in the identification task showed that these two variables were related (*r* = −0.64, *p* < 0.001). However, this correlation did no longer appear once the age of children was entered as a covariate in the analysis (*r* = −0.15, *p* > 0.05). Therefore, the relationship between the use of fingers and the identification performance of finger pattern was merely due to children's natural development.

### Enumeration task (Give-*N* task)

No overt finger counting was observed in the enumeration task and the analysis was carried out on the percentages of correct responses after the arcsin transformation was applied to the data. An ANOVA with the same design as before was carried out on the transformed data (Table 5). The main effect of School level was significant, *F*_(2, 57)_ = 24.98, η^2^_*p*_ = 0.47, *p* < 0.001, and showed that first graders (98%) were more successful than kindergartens (73%) and preschoolers (62%), *F*_(1, 57)_ = 24.76, η^2^_*p*_ = 0.30, *p* < 0.001. Moreover, there was a main effect of Numerosities, *F*_(2, 114)_ = 53.17, η^2^_*p*_ = 0.48, *p* < 0.001, showing that children were more successful with small (98%) than medium numerosities (76%), *F*_(1, 57)_ = 17.43, η^2^_*p*_ = 0.23, *p* < 0.001 and more successful with medium than large ones (57%), *F*_(1, 57)_ = 43.97, η^2^_*p*_ = 0.44, *p* < 0.001. Furthermore, the interaction between the two variables was significant, *F*_(4, 114)_ = 10.30, *p* < 0.001, η^2^_*p*_ = 0.27 and revealed that the effect of numerosities decreased as a function of school level.

**Table 5:** **Percentages of correct responses in the Give-*N* task as a function of School level and Numerosities of collection**.

	**Small**	**Medium**	**Large**
First Graders	100.00	98.33	95.00
Kindergartens	98.25	73.68	45.61
Preschoolers	96.67	56.67	31.67

Finally, a correlational analysis between the transformed percentages of spontaneous use of fingers in the counting task and the transformed percentages of correct responses in the Give-*N* task showed that these two variables were positively related (*r* = 0.59, *p* < 0.001). Importantly, this correlation was still significant when the age of participants was entered as a covariate (*r* = 0.25, *p* = 0.05). This attests that a part of performance in the Give-*N* is related to the frequency of spontaneous finger use in counting and not only to natural development. However, it is crucial to note that, on a more descriptive level, 5 children out of the 21 who scored the highest on the Give-*N* task were children who did not use their fingers in the picture counting task. This attests that using fingers to count is not a necessary stage for developing good verbal numerical abilities.

## General discussion

The aim of this study was to clarify the nature of the relationship between finger use and numerical processing. We were interested in determining whether finger use constitutes a necessary step for the development of numerical abilities. If finger use constitutes a necessary transient developmental stage between non-symbolic and symbolic numerical abilities, children who do not use their fingers for counting should not be able to succeed in numerical tasks. Less drastically, if finger use is rather a useful tool for the development of later numerical abilities, children who use their fingers more frequently should be more successful in numerical tasks than children who use their fingers more rarely. In order to determine which of these alternatives has to be retained, we assessed 4–7-year-old-children's performance on their spontaneous use of fingers in a counting task, on a finger numerical pattern recognition task and on an enumeration task (i.e., Give-*N* task). Then, and crucially for our purpose, we examined the correlations between the frequencies of finger use in the counting task with the performance on the other numerical tasks under study.

We showed that the percentages of spontaneous finger use in a counting task increased with school level. In fact, out of 20 children in each age group, only 1 preschooler and 3 kindergartens used their fingers, whereas 18 first graders used the finger strategy to solve the task. As expected, finger use was obviously a good strategy in this task because its frequency positively correlated with the percentages of correct responses in the task, even when the age was considered as a covariate. Moreover, it was virtually impossible for children who did not use their fingers to succeed in the task when large numerosities were concerned. This strong relationship between finger use and success in the task attests that covert use of fingers or other means to represent numbers were not implemented by children. Indeed, if unnoticed external aids have been used, such trials would also have been associated to correct responses and no correlation would have been observed. This indicates that the task we conceived is a good test in order to determine whether or not children would spontaneously implement a strategy based on finger counting. Furthermore, as already mentioned in the Introduction, the picture counting task is a powerful tool to assess finger use in children because it can reveal whether finger counting belongs to the child's strategy repertoire even after she or he has ceased to use fingers for calculations. Indeed, when children do not use their fingers to solve arithmetic problems, it is impossible to determine whether they do not need them any longer or whether they never have resorted to them. On the contrary, in the picture counting task, fingers are still required to succeed in the task. Then, children who do not use their fingers are necessarily children for whom this strategy is not available. We can therefore confidently conclude that children who do not use their fingers in the picture counting task do not correspond to children who relied on their fingers in previous stages of development.

Then, we showed that finger pattern comparison and finger pattern identification do not correlate with the frequency of finger use when the age of children is neutralized. Thus, it is not because children use their fingers more frequently in a counting task that it will help them to identify canonical and non-canonical finger patterns more accurately or more quickly than children who use their finger more rarely. Moreover, whatever their school level, children seem to be sensitive to the canonicity of configurations in the comparison task, despite the fact that preschoolers and kindergartens very rarely use their fingers to count. This attests that a mere observation or exposure to canonical configurations is sufficient to recognize them. Still, it is interesting to note that, as soon as the task requires more than a simple pattern processing, the sensibility to canonical patterns is no longer observed in younger children. It turns out that the comparison task can be performed on a pure perceptual basis because two patterns have simply to be judged as similar or different. Our results show that, whatever their age, children benefit from the familiarity of canonical configurations. Nevertheless, the identification task not only requires children to process the patterns but also to recognize and label them. Our results show that the matching between canonical patterns and verbal tags has not been strongly established in younger children.

Finally and crucially for our purpose, we showed that there is a correlation between the frequency of finger use in a counting task and the percentages of correct responses in an enumeration task (i.e., Give-*N* task). Because children who use their fingers more frequently are mainly the oldest children in our study, we had to ensure that this correlation was not only due to natural development. For this purpose, we entered the age of children as a covariate in our analysis and showed that the correlation was still significant. Therefore, we can conclude quite confidently that finger use is a useful tool for the development of symbolic, at least verbal, numerical abilities. However, we also showed that despite this correlation, some children who do not use their fingers in the counting task are able to perform optimally in the enumeration task. Then, and in accordance to Crollen et al. (2011a,b), our study suggests that fingers are not a necessary tool for the development of counting abilities. In others words, using fingers could constitute a beneficial step for an efficient transition from non-symbolic to symbolic numerical skills. Yet, succeeding in manipulating symbolic numerical representations is possible without this transitional stage. Within the embodied cognition framework, our results suggest that the integration of motor input during early finger counting and finger calculation can help children in their later numerical acquisitions. However, this integration does not seem necessary to develop accurate symbolic numerical representations.

Overall and interestingly, our pattern of results show that the use of finger in a counting task is related to the ability of children in a dynamic enumeration task but not to static tasks involving recognition and comparison of finger patterns. Therefore, it could be that the link between finger and numbers remain circumscribed to counting tasks and do not extent to static finger montring situations.

Beside theoretical considerations, our results could have implications concerning educational issues, assessment of children with numerical difficulties and remediation of numerical skill impairments. Indeed, it could be fruitful to more explicitly encourage children in using their fingers and establishing the link between fingers and numerosities. This could help them in constructing stable numerical representations in strengthening the link between concrete and analog representations and verbal symbolic codes (Fayol and Seron, 2005, but see Brissiaud, 2013 for a different point of view). Moreover, our study confirms the relevance of evaluating finger use in early neuropsychological assessments of numerical skills.

### Conflict of interest statement

The authors declare that the research was conducted in the absence of any commercial or financial relationships that could be construed as a potential conflict of interest.

## Figures and Tables

**Table A1:**
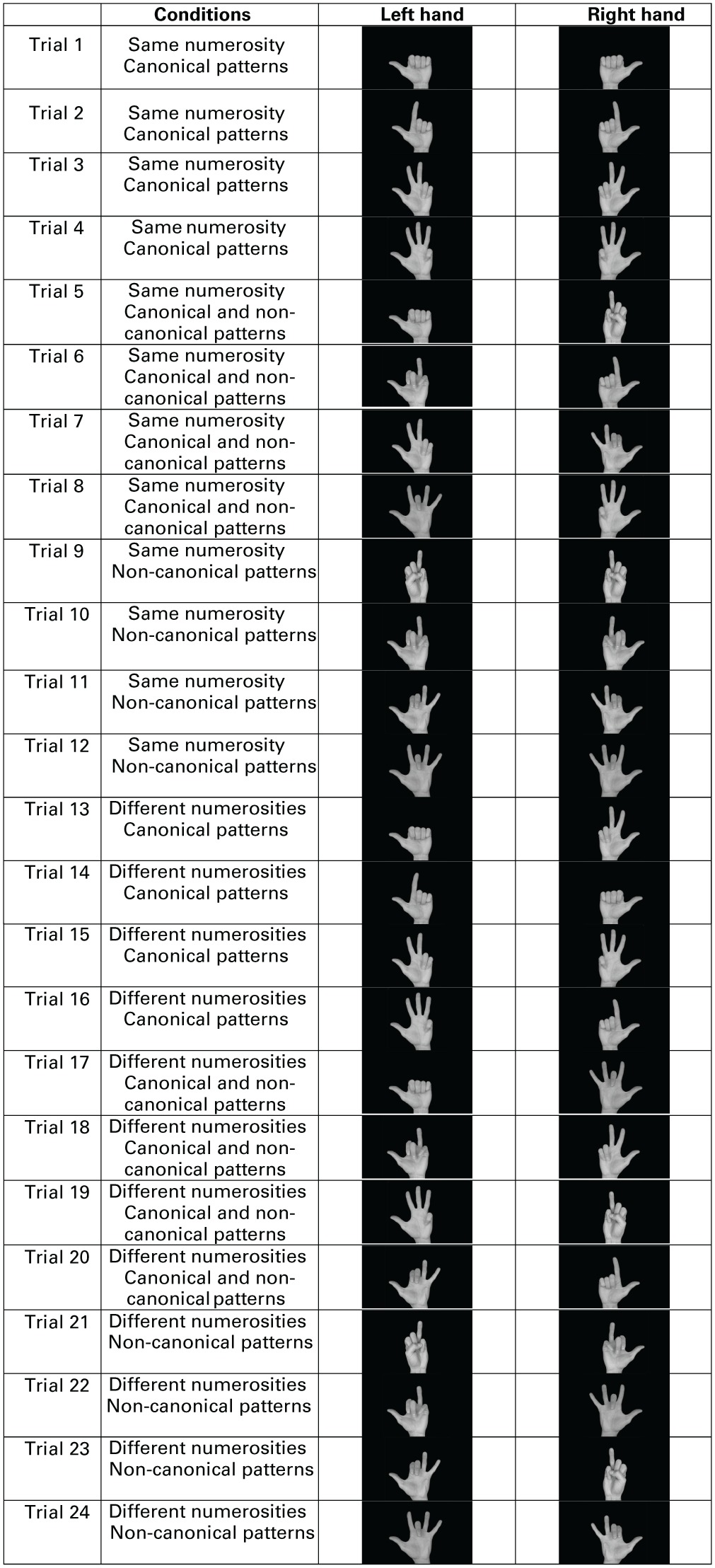
**List of the 24 trials presented in the comparison of finger numerical pattern (adapted from Noël, 2005 and Gracia-Bafalluy and Noël, 2008)**.

**Table A2:**
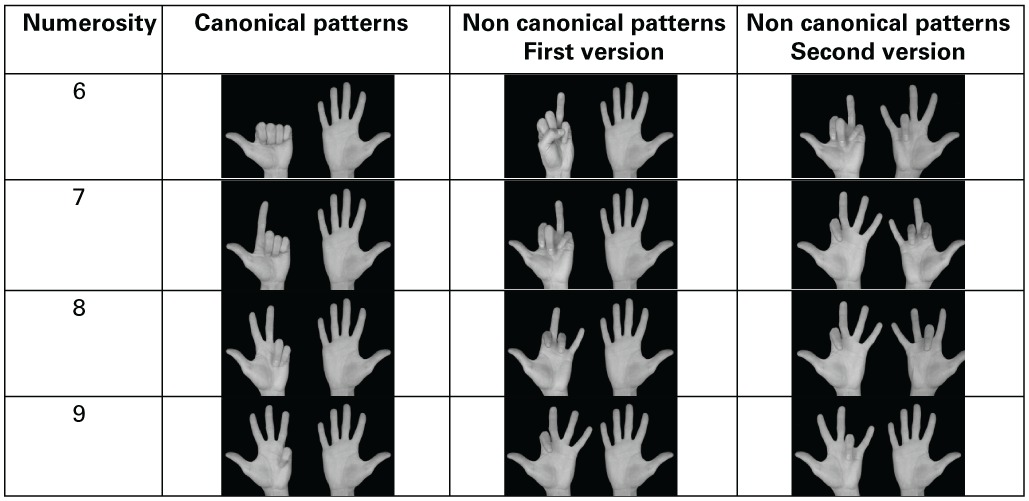
**Additional pictures presented in the identification of finger numerical pattern task (adapted from Noël, 2005 and Gracia-Bafalluy and Noël, 2008)**.
